# Construction of UiO-66/Bi_4_O_5_Br_2_ Type-II Heterojunction to Boost Charge Transfer for Promoting Photocatalytic CO_2_ Reduction Performance

**DOI:** 10.3389/fchem.2021.804204

**Published:** 2021-12-13

**Authors:** Dongsheng Li, Bichen Zhu, Zhongti Sun, Qinqin Liu, Lele Wang, Hua Tang

**Affiliations:** ^1^ School of Materials Science and Engineering, Jiangsu University, Zhenjiang, China; ^2^ School of Environmental Science and Engineering, Qingdao University, Qingdao, China

**Keywords:** UiO-66, Bi_4_O_5_Br_2_, photocatalytic, CO_2_ reduction, type-II heterojunction

## Abstract

One of the basic challenges of CO_2_ photoreduction is to develop efficient photocatalysts, and the construction of heterostructure photocatalysts with intimate interfaces is an effective strategy to enhance interfacial charge transfer for realizing high photocatalytic activity. Herein, a novel UiO-66/Bi_4_O_5_Br_2_ heterostructure photocatalyst was constructed by depositing UiO-66 nanoparticles with octahedral morphology over the Bi_4_O_5_Br_2_ nanoflowers assembled from the Bi_4_O_5_Br_2_ nanosheets *via* an electrostatic self-assembly method. A tight contact interface and a built-in electric field were formed between the UiO-66 and the Bi_4_O_5_Br_2_, which was conducive to the photo-electrons transfer from the Bi_4_O_5_Br_2_ to the UiO-66 and the formation of a type-II heterojunction with highly efficient charge separation. As a result, the UiO-66/Bi_4_O_5_Br_2_ exhibited improved photocatalytic CO_2_ reduction performance with a CO generation rate of 8.35 μmol h^−1^ g^−1^ without using any sacrificial agents or noble co-catalysts. This work illustrates an applicable tactic to develop potent photocatalysts for clean energy conversion.

## 1 Introduction

The high-speed increase of carbon dioxide (CO_2_) concentration in the atmosphere has led to serious global warming and environmental problems (Scott and Geden, 2018; Prasad et al., 2020; Tang et al., 2020; Xu et al., 2020; Liu et al., 2021a). Investigating reliable strategies which can convert CO_2_ into usable fuel can guide the healthy development of society. Among the numerous proposals for CO_2_ conversion, solar photocatalysis that can efficiently convert CO_2_ into useful chemical intermediates and chemical raw material (carbon monoxide, CO) has been appraised as a potential strategy that can overcome global warming as well as meet the demand for renewable fuels (Shen et al., 2019; Liu et al., 2020a). Therefore, the development of highly efficient photocatalysts plays a considerable role in the future practical application of photocatalytic CO_2_ conversion (Liu, 2016; Wu et al., 2019; Wang et al., 2021a; Wang et al., 2021b).

In recent years, metal-organic framework (MOF) based semiconductors demonstrating attractive characteristics such as high surface area and a well-defined and adjustable porous structure are considered as a promising material for photocatalytic applications of organic pollutants degradation, O_2_ production, H_2_ production, N_2_ fixation, and CO_2_ reduction (Hirakawa et al., 2016; Ling et al., 2018; Dong et al., 2019; Mukhopadhyay et al., 2021; Peña-Velasco et al., 2021). Among the assorted MOF-based photocatalysts, UiO-66 is a classic Zr-based MOF material, and it has high thermal stability, unsaturated open metal sites, hygroscopic CO_2_ binding sites, and superior CO_2_ adsorption capacity. Therefore, UiO-66 is considered a promising candidate for application in photocatalytic CO_2_ reduction (Kim et al., 2015; Wang et al., 2018a). For example, Yamani et al. prepared UiO-66-NH_2_ based frameworks using a hydrothermal method and found it presented photocatalytic CO_2_ reduction activity with the products of CO, CH_4_, and HCOO^−^ (Zeama et al., 2020). However, the pure UiO-66 usually demonstrates a fast recombination rate of electron holes and poor photocatalytic CO_2_ reduction activity (Peng et al., 2020). Recently, some tactics, such as replacing the original ligands (Zhang et al., 2021), depositing precious metals or other co-catalysts on the surface (Zhang et al., 2020), and doping other metal elements to form new coordination bonds (Yang et al., 2017) have been investigated to ameliorate the performance of UiO-66. Coupling UiO-66 with another photocatalytic semiconductor to construct a heterostructure composite can adjust the electronic transfer path and achieve high separation efficiency of photogenerated charges, which has been deemed as one of the most promising procedures to elevate the photocatalytic performance (Su et al., 2017).

Choosing a suitable semiconductor is one of the key points to construct heterojunctions with UiO-66. Among the reported semiconductors, bismuth oxyhalide BiOX (X = Cl, Br, and I) consisting of a [Bi_2_O_2_] layer interlaced by double layers of halogen atoms is a typical layered semiconductor, and it illustrates unique advantages of being chemically stable, non-toxic, and corrosion-resistant (Jin et al., 2019; Ren et al., 2020). It is noted that the band energy structure of BiOX can be controlled by adjusting the Bi content in bismuth oxybromide material. For instance, Ye et al. have fabricated the Bi_4_O_5_Br_2_ nanoflowers via a bismuth-rich strategy and found that Bi_4_O_5_Br_2_ nanoflowers with high Bi ratio owned smaller band gap and better CO_2_ photoreduction activity compared to BiOBr (Ye et al., 2016). Accordingly, the BiOX with adjustable band structure seems to be a promising choice to match with UiO-66 to construct heterojunction photocatalysts for the application of CO_2_ reduction.

**Scheme 1:**
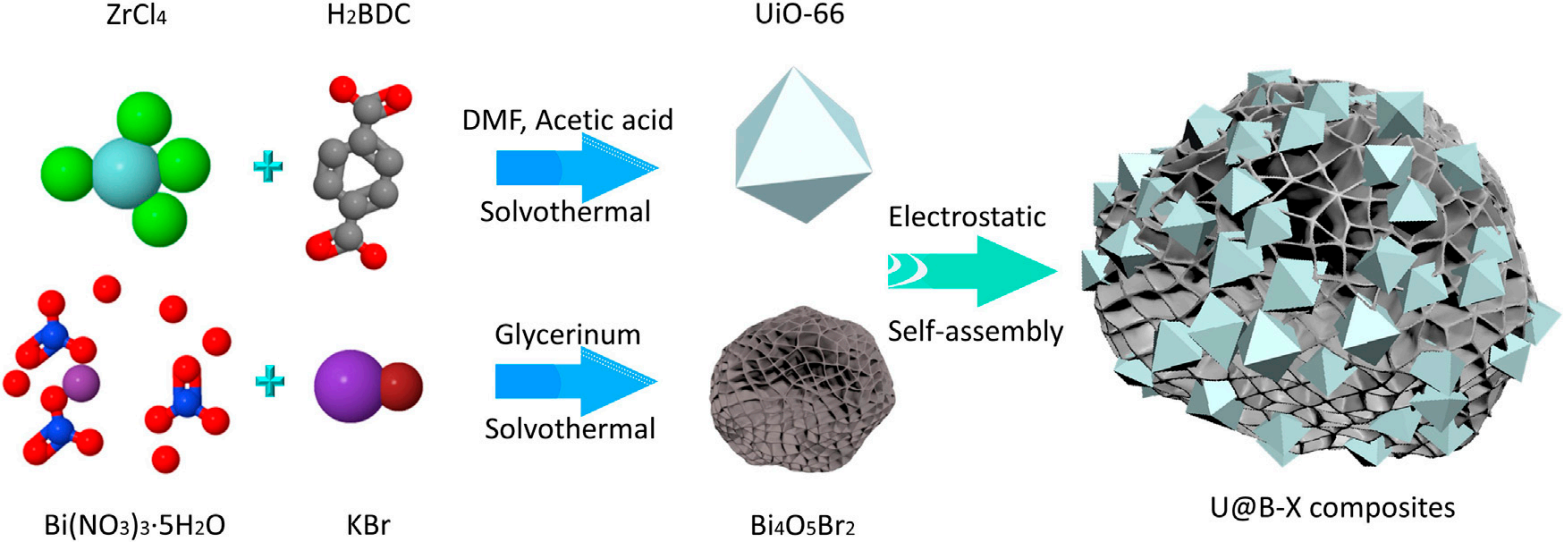
Schematic depiction of preparation of U@B-X composites.

In this study, a novel UiO-66/Bi_4_O_5_Br_2_ (U@B) heterojunction was fabricated *via* an electrical self-assembly method by wrapping the UiO-66 nanoparticles over the Bi_4_O_5_Br_2_ nanoflowers (Scheme 1). The tight contact interface and matched energy band structure between UiO-66 and Bi_4_O_5_Br_2_ could accelerate the charge separation to promote CO_2_ reduction property. The UiO-66@Bi_4_O_5_Br_2_ hybrid can effectively produce CO at a rate of 8.35 μmol h^−1^ g^−1^ under full-spectrum light irradiation without using any noble co-catalysts or sacrificial agents. This tactic of constructing MOFs-based heterojunction provides a new way to design efficient photocatalysts with CO_2_ reduction activity.

## 2 Experimental

### 2.1 Fabrication of the UiO-66/Bi_4_O_5_Br_2_ Composite

For the preparation of pure UiO-66, 1.0 mmol ZrCl_4_ (233.04 mg) and 1.0 mmol terephthalic acid (H_2_BDC 166.13 mg) were dispersed into 25 ml DMF solution and sonicated for 10 min, respectively. After that, 3.6 ml acetic acid was mixed into the above solution in a 100 ml Teflon-lined autoclave and sonicated for 10 min and then heated at 120°C for 24 h. After that, the collection was centrifugated, washed, and dried overnight at 60°C to obtain the UiO-66. For the Bi_4_O_5_Br_2_, 2 mmol KBr, and 2 mmol Bi(NO_3_)_3_·5H_2_O were dispersed into 20 ml glycerol and stirred for 10 min, respectively. Then, the KBr solution and Bi(NO_3_)_3_·5H_2_O solution were transferred into a 50 ml Teflon-lined autoclave and kept at 160°C for 16 h. After cooling down, the precipitate was washed and dried to obtain Bi_4_O_5_Br_2_.

The UiO-66/Bi_4_O_5_Br_2_ composites were prepared by an electronic assembly technique. Typically, 50 mg Bi_4_O_5_Br_2_ with a positive zeta potential of 35.9 mV (Supplementary Figure S1) and different amounts of UiO-66 with a negative zeta potential of −20.1 mV (Supplementary Figure S1) were dispersed into 50 ml deionized water, respectively, and the above two solutions were mixed together and then sonicated for 60 min to prepare the UiO-66@Bi_4_O_5_Br_2_ composite. The adding amount of the UiO-66@ was 5, 10, 15 mg, respectively, and the corresponding UiO-66@Bi_4_O_5_Br_2_ samples were named U@B-5, U@B-10, and U@B-15, respectively.

### 2.2 Characterization Techniques

The structure, morphology, composition, and zeta potential were tested using powder X-ray diffraction (XRD, D/Max-2550, Rigaku), field emission scanning electron microscopy (FE-SEM, JXA-840A, JEOL), high-resolution transmission electron microscopy (HR-TEM, JEM-100CX II, JEOL), X-ray photoelectron spectroscopy (XPS, PHI ESCA-5000C, Perkin Elmer), and a ZS90 (Malvern Panalytical) zeta sizer, respectively. The light adsorption, Fourier transform infrared spectrum (FT-IR), Raman spectrum and transient fluorescence absorption spectroscopy were studied by the UV–vis diffuse reflectance spectra (DRS, UV2550 spectrophotometer), Nexus 870 spectrometer, DXR spectrometer, and femtosecond transient absorption spectrometer (Helios fire, Ultrafast System), respectively. The S_BET_ and pore size distribution of the powders were measured on an ASAP 2020 HD88 (United States). The photocurrents, electrochemical impedance spectroscopy (EIS) profiles, and Mott–Schottky plots were tested via a CHI660B electrochemical workstation.

### 2.3 Photocatalytic Performance Measurements

The photocatalytic CO_2_ reduction experiments were measured in an enclosed quartz reactor under Xe lamp irradiation (Perfectlight, PLS-SXE300+) and the reaction temperature was controlled at 5°C by HJDC-0506 instrument to prevent thermal catalytic effects. Typically, 30 mg of U@B-X sample was dispersed in 50 ml of deionized water with no sacrificial agent by stirring for 10 min and then transformed into the enclosed quartz reactor. Then, the reaction container was filled with high purity CO_2_ gas (99.995%) and the gaseous products were tested by the GC-2014C gas chromatography (GC, Shimadzu Technologies) equipped with a thermal conductivity detector (TCD) and two flame ionization detectors (FIDs) for the analysis of CO. For the stability measurement, the reactor was re-filled with CO_2_ (80Kpa) and then tested under the same condition described above.

### 2.4 Photoelectrochemical Measurements

The photocurrents, electrochemical impedance spectroscopy (EIS) profiles, Mott–Schottky plots, and Linear sweep voltammetry (LSV) were tested *via* a CHI-760E electrochemical workstation.

Photocurrent measurements of samples were carried out using a three electrodes electrochemical workstation with Ag/AgCl as reference electrode, Pt wire as the counter electrode, and the catalysts as the working electrode using a 75 W 365 nm LED lamp as the light source. The working electrode was prepared with 5 mg catalysts, 250 μL ethanol, 250 μL ethylene glycol, and 40 μL Nafion solution (5 wt%) dip-coated on the FTO conducting glass. Mott-Schottky plot of UiO-66 and Bi_4_O_5_Br_2_ were measured without light irradiation at 200 Hz, 400Hz, and 600 Hz frequencies. EIS measurements were tested in the frequency range of 1,000 kHz–0.01 Hz at 0.8 V. LSV measurements were performed by sweeping the potential from 0 to 1.2 V Na_2_SO_4_ aqueous solution (0.1 M, pH = ca. 7) was used as the supporting electrolyte.

## 3 Results and Discussions

**FIGURE 1:**
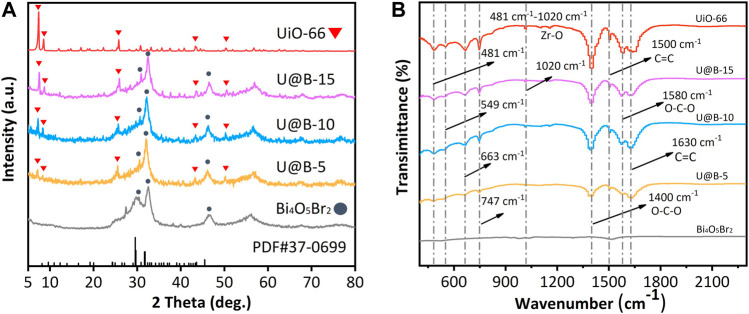
**(A)** XRD patterns of UiO-66, Bi_4_O_5_Br_2_ and U@B-X, **(B)** FTIR spectra of UiO-66, Bi_4_O_5_Br_2_, and U@B-X.

The composition of different samples was characterized by XRD. Pure Bi_4_O_5_Br_2_ shows a series of diffraction peaks at 24.4, 27.4, 29.6, 32.6, and 46.2^o^, corresponding to (31-1), (212), (11-3), (020), and (422) planes of the standard monoclinic Bi_4_O_5_Br_2_ (PDF #37-0699); while the diffraction peaks of UiO-66 are consistent with that reported in the literature (Figure 1A) (Dong et al., 2015), indicating the successful synthesis of Bi_4_O_5_Br_2_ and UiO-66. Both peaks corresponding to the Bi_4_O_5_Br_2_ and UiO-66 are observed in Bi_4_O_5_Br_2_/UiO-66 hybrid, no obvious change is observed in the crystal structure after the combination of UiO-66 and Bi_4_O_5_Br_2_, indicating successful fabrication of the Bi_4_O_5_Br_2_/UiO-66 hybrid (Figure 1A). The peak intensity of UiO-66 in the Bi_4_O_5_Br_2_/UiO-66 hybrid increases when UiO-66 content increases. The different functional groups of the UiO-66 and the Bi_4_O_5_Br_2_/UiO-66 hybrid were studied via FTIR technique (Figure 1B). For pure UiO-66, a series of peaks representing asymmetrical stretching of Zr-(OC) and different bending vibration modes from ligands are observed between 400 and 1,020 cm^−1^ (Bargozideh et al., 2020). The two peaks at 1,400 and 1,580 cm^−1^ represent the O-C-O symmetric and asymmetric tensile vibrations of the carboxyl groups of the ligands of the terephthalic acid part. The peaks at 1,500 and 1,630 cm^−1^ are attributed to the C=C vibration mode of the H_2_BDC ligands (Ahmadipouya et al., 2021). The weak peak of C=C for the Bi_4_O_5_Br_2_ sample is caused by the adsorption of carbon impurities (glycerin) on the surface during the synthetic process. The Bi_4_O_5_Br_2_/UiO-66 hybrids demonstrate almost the same spectra as that of pure UiO-66, indicating that the coupling UiO-66 to Bi_4_O_5_Br_2_ would not change the functional group structure of UiO-66. In addition, Supplementary Figure S2 shows the Raman spectra of UiO-66, Bi_4_O_5_Br_2,_ and Bi_4_O_5_Br_2_/UiO-66 composite. For the UiO-66, the peaks at 1,610, 1,440, and 1,420 cm^−1^ correspond to the C=C stretching and coordination of Zr(IV) ions with the O-C-O symmetric stretching, respectively, while the low-frequency bands at 1,140 and 860 cm^−1^ can be assigned to the deformation band of the benzoic acid group (Bariki et al., 2020). For the Bi_4_O_5_Br_2_, two Raman peaks of A_1g_ internal and E_1g_ internal Bi-Br stretching modes are found at 108 and 156 cm^−1^, respectively (Xu et al., 2021). Both Raman bands belonging to UiO-66 and Bi_4_O_5_Br_2_ are observed in the U@B-X composites. The XRD, FTIR, and Raman results further proved the successfully synthesized of Bi_4_O_5_Br_2_/UiO-66 hybrids.

**FIGURE 2:**
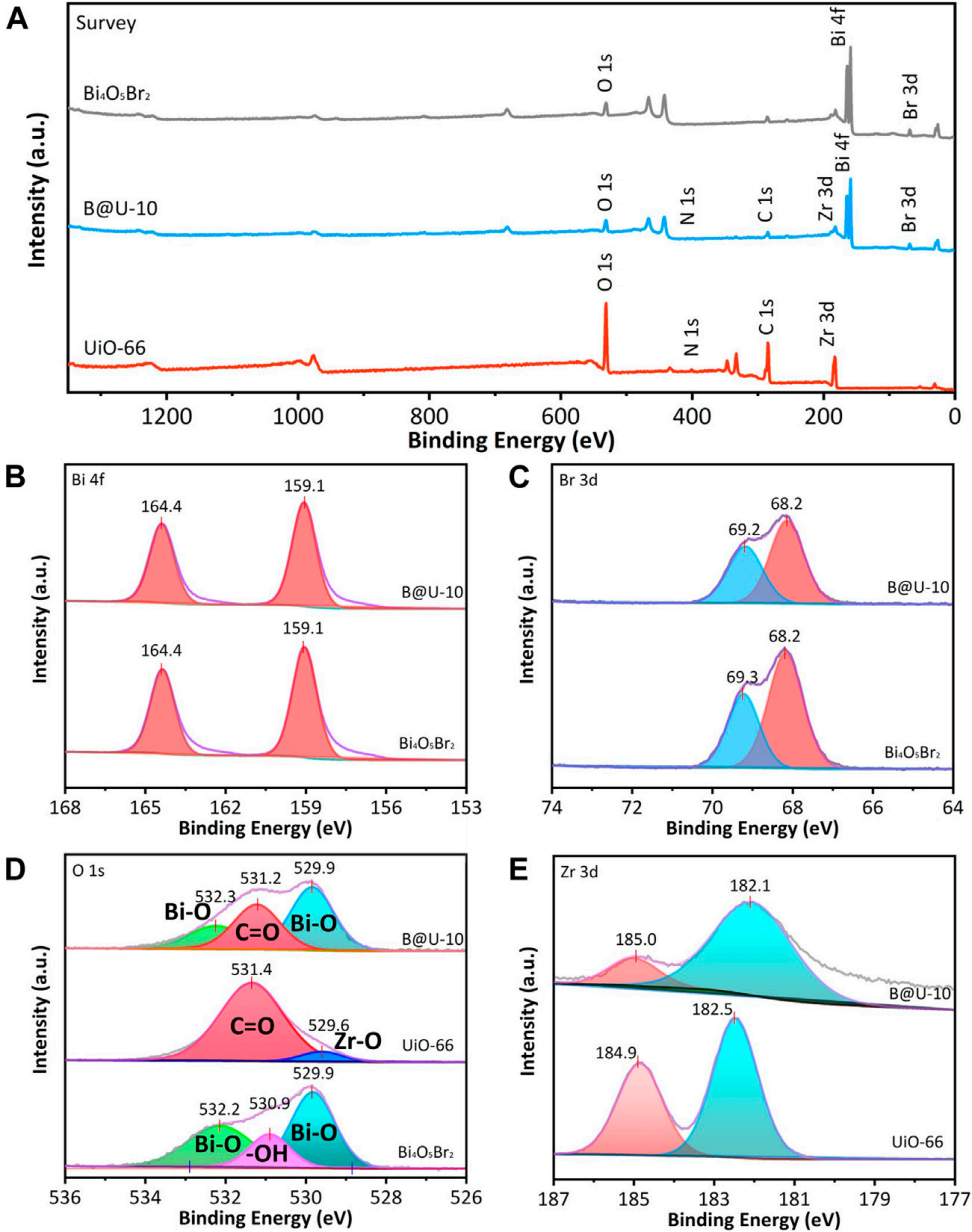
XPS survey spectra **(A)**, high-resolution spectra of **(B)** Bi 4f, **(C)** O 1s, **(D)** Br 3d, and **(E)** Zr 3d for UiO-66, Bi_4_O_5_Br_2_, and U@B-10.

The survey XPS spectra show that the Bi_4_O_5_Br_2_/UiO-66 composite mainly contains O, C, Zr, Bi, and Br elements (Figure 2A), indicating the UiO-66 and Bi_4_O_5_Br_2_ are successfully integrated *via* the self-assembly method. The high-resolution spectrum of Bi 4f of the Bi_4_O_5_Br_2_ illustrates doublet peaks representing Bi 4f7/2 (159.1 eV) and Bi 4f5/2 (164.4 eV), respectively, implying that Bi atoms are Bi^3+^ in pure Bi_4_O_5_Br_2_ (Figure 2B) (Ye et al., 2016). For the Bi 4f in U@B-10 composite (Figure 2B), the Bi 4f spectrum also can be divided into two peaks at 159.1 and 164.4 eV, and no difference is observed compared to the pure Bi_4_O_5_Br_2_, indicating that Bi^3+^ can be maintained. The Br 3d spectrum of Bi_4_O_5_Br_2_ can be divided into two peaks at 68.2 and 69.3 eV, representing Br 3d5/2 and Br 3d3/2, respectively (Figure 2C) (Bai et al., 2019). Besides, the peak position of Br 3d in U@B-10 is still located at 68.2 and 69.2 eV, respectively, revealing the same status of the Br in U@B-10 and Bi_4_O_5_Br_2_. The O1s spectrum of UiO-66 in Figure 2D is decomposed into two peaks of Zr-O (529.6 eV) and C=O groups (531.4 eV), respectively (Cao et al., 2021). The peaks at 529.9 and 532.2 eV in Bi_4_O_5_Br_2_ can be ascribed to the Bi-O band, while the peak of 530.9 eV stands for the surface hydroxyl groups (Chen et al., 2021; Hong et al., 2020). Figure 2E shows the spectra of Zr 3d, two strong peaks can be observed at 182.5 and 184.9 eV of UiO-66, corresponding to the Zr 3d5/2, and Zr 3d3/2, respectively (Ding et al., 2017). The XPS results evidenced the successful preparation of the Bi_4_O_5_Br_2_/UiO-66 composite.

**FIGURE 3:**
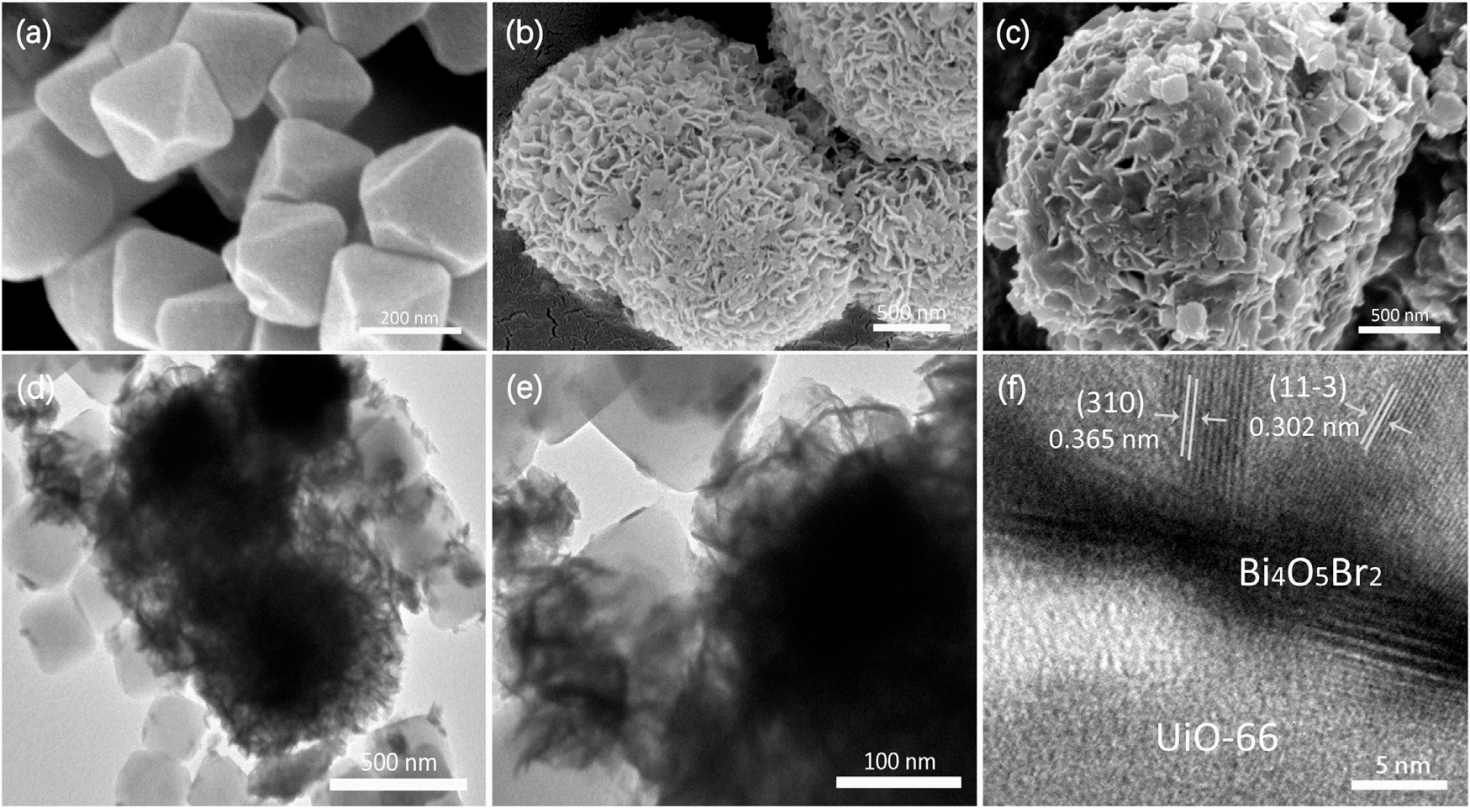
FESEM images of **(A)** UiO-66, **(B)** Bi_4_O_5_Br_2_, **(C)** U@B-10, **(D–E)** TEM of U@B-10, and **(F)** HRTEM of U@B-10.

The SEM image of UiO-66 is illustrated in Figure 3A, exhibiting a relatively uniform octahedral prism morphology with an average size of 200 nm. The pure Bi_4_O_5_Br_2_ shows hierarchical and stereoscopic flower morphology, which is made up of small nanosheets (Figure 3B). For the U@B-10 hybrid, the UiO-66 octahedral prism is wrapped over the Bi_4_O_5_Br_2_ stereoscopic flowers (Figures 3C,D). The corresponding energy-dispersive X-ray spectroscopy (EDX) element analysis (Supplementary Figure S3) displays the coexistence of Bi, Br, O, and Zr elements at the selected area of U@B-10, further proving the effective hybridization of UiO-66 and Bi_4_O_5_Br_2_. Furthermore, an intimate interface and the crystal boundary can be observed between the UiO-66 and Bi_4_O_5_Br_2_ (Figures 3E,F), which also proves the formation of a heterostructure. Figure 3F clearly shows the polycrystalline properties of Bi_4_O_5_Br_2_, and the (11-3) and (310) lattice planes of Bi_4_O_5_Br_2_ with lattice edges of 0.30 and 0.36 nm, respectively, are also observed in the U@B-10 hybrid. The Brunner−Emmet−Teller (BET) surface area of UiO-66, Bi_4_O_5_Br_2_, and U@B-10 composite is displayed in Supplementary Table S1, and the U@B-10 composite displays a much larger BET surface area (211.12 m^2^ g^−1^) and pore volume (0.14 m^3^ g^−1^) than that of Bi_4_O_5_Br_2_. From the TEM images of the samples can find that part of the surface of UiO-66 was covered by Bi_4_O_5_Br_2_ after combination, which decreased the surface area of the composite. The U@B-10 sample owns the highest average pore size (5.92 nm), which is beneficial for exposing more active sites thereby enhancing the photocatalytic activity.

**FIGURE 4:**
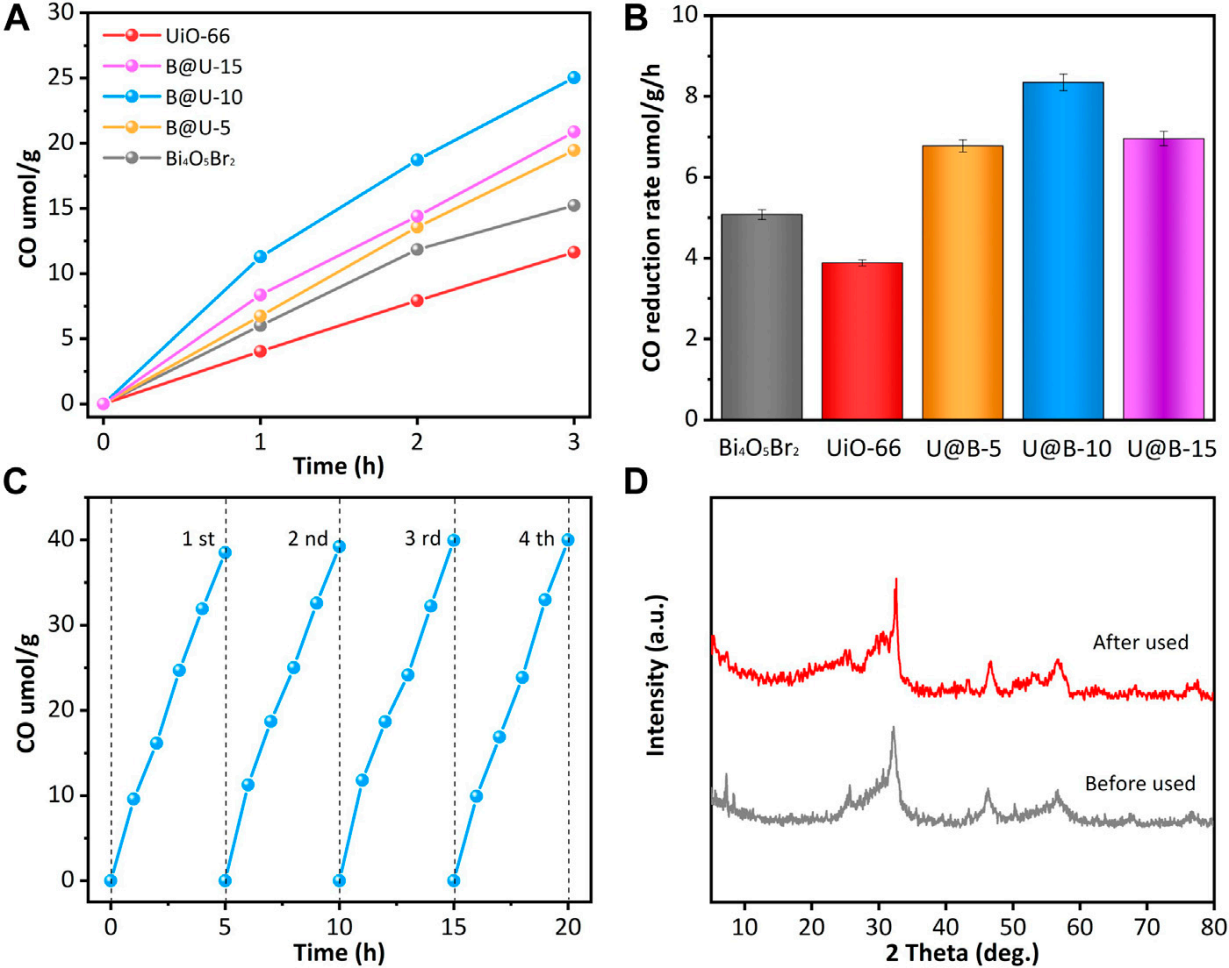
**(A)** Photocatalytic CO_2_ reduction and **(B)** CO_2_ reduction rates of different samples; **(C)** stability test of U@B-10 and **(D)** the XRD patterns of U@B-10 before and after cycles.

The photocatalytic CO_2_ reduction performances were investigated (Figures 4A,B). Pure UiO-66 and Bi_4_O_5_Br_2_ show rather low CO_2_ reduction performance. Pure UiO-66 and Bi_4_O_5_Br_2_ present the photocatalytic CO production rate of 3.88 umolg^−1^h^−1^ and 5.08 umolg^−1^ h^−1^, respectively. Compared with pure Bi_4_O_5_Br_2_ and UiO-66, coupling the UiO-66 octahedron on Bi_4_O_5_Br_2_ can effectively improve the CO_2_ reduction efficiency. U@B-X samples demonstrate enhanced CO_2_ reduction performance: the photocatalytic CO reduction rate increases in the following order UiO-66 < Bi_4_O_5_Br_2_ < U@B-5 < U@B-15 < U@B-10. The low CO_2_ reduction of the U@B-15 may be due to the excessive deposition of UiO-66 on Bi_4_O_5_Br_2_ impeding the light absorption of Bi_4_O_5_Br_2_, thus inhibiting the generation of photogenerated carriers and reducing the transport of carriers. The optimal U@B-10 showed the strongest CO_2_ reduction rate of 8.35 umolg^−1^h^−1^, which is 2.15 times of pure UiO-66 and 1.64 times of pure Bi_4_O_5_Br_2._ It is noted that the activity of the U@B-10 is among the best reports for photocatalytic CO_2_ reduction (Supplementary Table S2). The stability is an important factor in the practical application of high-efficiency photocatalyst, therefore, CO_2_ reduction of U@B-10 was carried out four times under the full spectrum light (Figure 4C), and the CO production was calculated every 5 h. It can be seen that the CO production remained stable after every cycle and the crystalline structure preserved its original state (Figure 4D). All the results above indicate that the sample had excellent stability.

**FIGURE 5:**
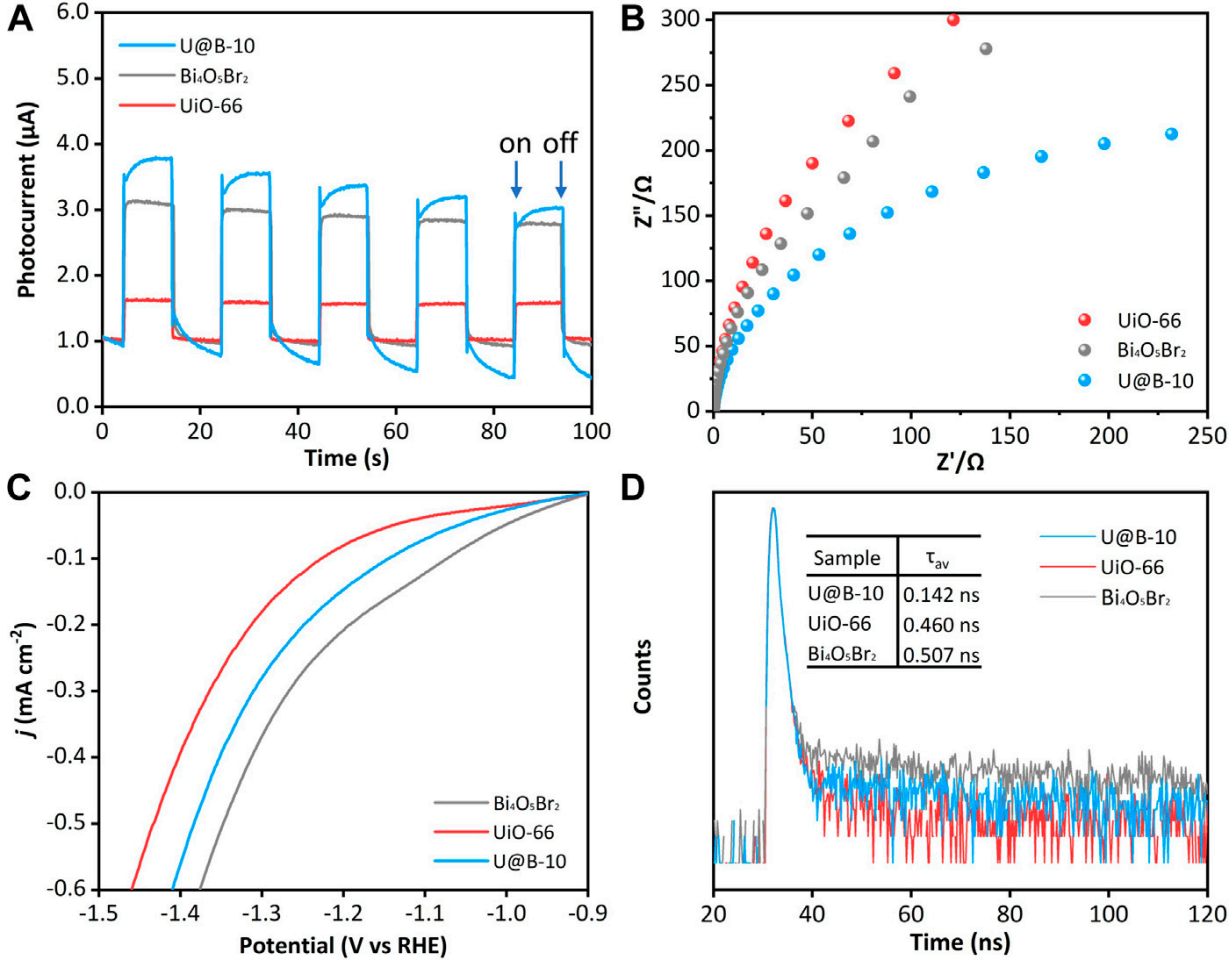
**(A)** Transient photocurrent spectra, **(B)** EIS spectra, **(C)** LSV curves, **(D)** TRPL of Bi_4_O_5_Br_2_, and U@B-10.

The charge separation behavior was investigated to explore the reason for the enhanced photocatalytic CO_2_ reduction performance. Figure 5A displays that the intensity of transient photocurrent of the UiO-66 is very low, while the U@B-10 composite demonstrates the highest transient photocurrent density compared to that of the UiO-66 and Bi_4_O_5_Br_2_, indicating that the U@B-10 composite owns the fastest separation efficiency of photogenerated carriers (Liu et al., 2020b; Liu et al., 2020c). The EIS was also used to characterize the interface charge carrier transport capacity. As shown in Figure 5B, the EIS radius of the U@B-10 composite is smaller than that of pure Bi_4_O_5_Br_2_ and UiO-66, indicating that U@B-10 composite presents lower charge transfer resistance and higher separation rate (Liu et al., 2020d; Wang et al., 2020). Furthermore, according to the linear sweep voltammetry (LSV) analysis (Figure 5C), U@B-10 shows a lower overpotential and Tafel slope (Supplementary Figure S4) than that of pure UiO-66, indicating that the introduction of Bi_4_O_5_Br_2_ can decrease the overpotential and promote CO_2_ reduction (Liu et al., 2016; Peng et al., 2021). Time-resolved PL spectra were performed (Figure 5D). The average lifetime of U@B-10 is shorter (0.142 ns) compared with Bi_4_O_5_Br_2_ (0.507 ns) and UiO-66 (0.460 ns), indicating that the U@B-10 heterojunction demonstrates faster transmission speed of the photogenerated carriers (Tang et al., 2019).

**FIGURE 6:**
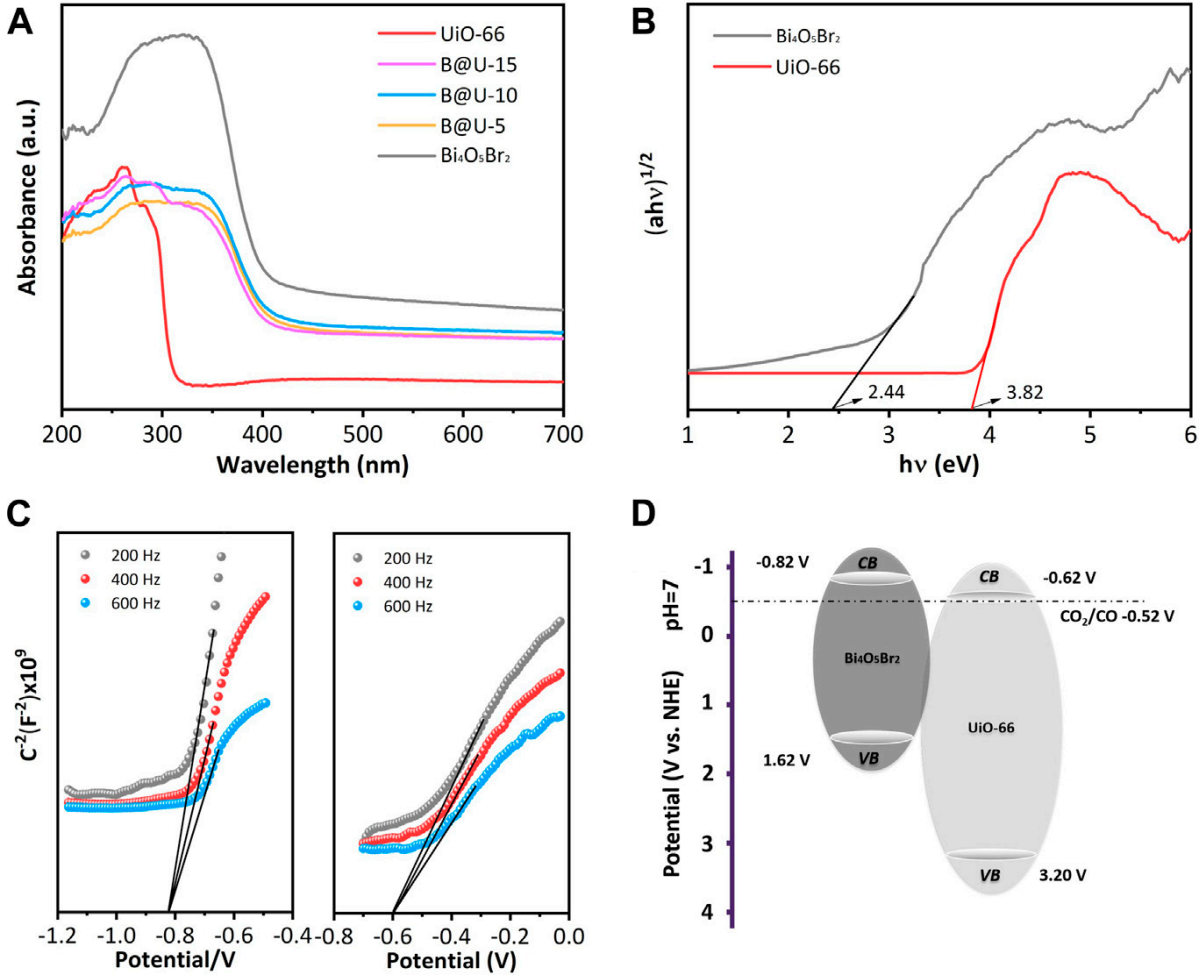
**(A)** UV–vis spectra, **(B)** Tauc plots, **(C)** MS plots, **(D)** band diagrams of UiO-66, and Bi_4_O_5_Br_2_.

Figure 6A reveals the UV-Vis spectra of the UiO-66, Bi_4_O_5_Br_2_, and U@B-X composites. Due to the Zr-_OXO_ cluster and the charge transfer transition between the ligand and Zr (IV) (Yu et al., 2020), UiO-66 exhibits a typical absorption edge at 345 nm (Wang et al., 2016). The Bi_4_O_5_Br_2_ demonstrates an obvious absorption edge at 440 nm. The band gap energies of UiO-66 and Bi_4_O_5_Br_2_ are calculated to be 3.82 and 2.44 eV, respectively (Figure 6B). According to the Mott-Schottky (MS) diagram (Figure 6C), the conduction band positions of the UiO-66 and Bi_4_O_5_Br_2_ are slated to be −0.62 and −0.82 V, respectively (Figure 6D) (Chai et al., 2018; Wei et al., 2021).

**FIGURE 7:**
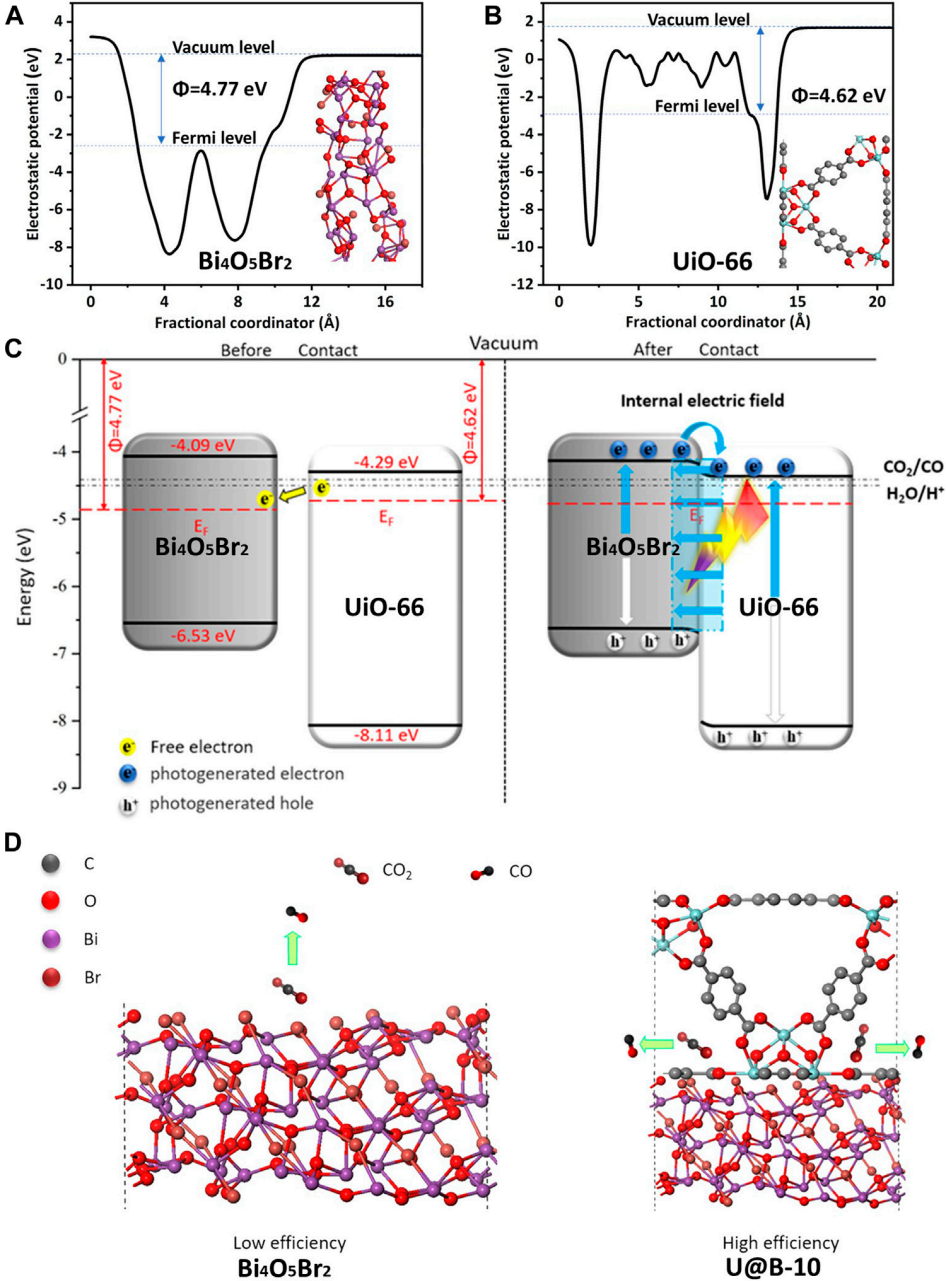
The calculated work functions **(A)** (11-3) plane of Bi_4_O_5_Br_2_ and **(B)** (100) plane of UiO-66; **(C,D)** the proposed photocatalytic mechanism of UiO-66/Bi_4_O_5_Br_2_ composite.

Moreover, the work function, which can reveal electron transfer in the heterostructure, was also calculated using the density functional theory (DFT) calculation (Liu et al., 2021b). Figures 7A,B shows that the work functions of Bi_4_O_5_Br_2_ (11-3) and UiO-66 (100) are 4.09 and 4.29 eV, respectively, indicating that the Bi_4_O_5_Br_2_ (11-3) surface Fermi levels is lower than that of UiO-66 (100) surface. Based on the Mott-Schottky curve, Bi_4_O_5_Br_2_, and UiO-66 are n-type semiconductors, and their Fermi energy levels are close to the conduction band. Since the Fermi energy level of Bi_4_O_5_Br_2_ is lower than that of UiO-66, the electrons of UiO-66 will transfer to Bi_4_O_5_Br_2_ until their Fermi energy levels reach equilibrium, and a built-in electric field is formed (Wang et al., 2018b; Tao et al., 2021). Accordingly, a preliminary charge transfer mechanism of U@B-X photocatalyst is proposed (Figures 7C,D). The UiO-66 and Bi_4_O_5_Br_2_ have a good matching band structure to form a type-II heterojunction. Based on the work functions of Bi_4_O_5_Br_2_ (11-3) and UiO-66 (100), the electrons tend to migrate from UiO-66 to Bi_4_O_5_Br_2_ forming an internal electric field. Driven by the internal electric field, the photogenerated electrons on the CB of Bi_4_O_5_Br_2_ are more easily transferred to the CB of UiO-66 to participate in the CO_2_ reduction reaction while the holes on the VB of UiO-66 transferred to the VB of Bi_4_O_5_Br_2_ (Wei et al., 2018; Kuang et al., 2021).

## 4 Conclusion

In summary, a series of UiO-66@Bi_4_O_5_Br_2_ photocatalysts were prepared via assembling UiO-66 octahedral on Bi_4_O_5_Br_2_ nanoflower using a simple electrostatic self-assembly method. The UiO-66@Bi_4_O_5_Br_2_ hybrids exhibited elevated photocatalytic CO_2_ reduction activity under full-spectrum light illumination without any sacrificial agent, the optimal sample (U@B-10 hybrid) showed the highest CO conversion rate of 8.35 μmol h^−1^ g^−1^, which was 1.64 and 2.15 times higher than pure Bi_4_O_5_Br_2_ and UiO-66, respectively. A possible mechanism of enhanced activity was supposed that UiO-66 and Bi_4_O_5_Br_2_ demonstrated well-matched energy band structures and an intimate interface, leading to the construction of a type-II heterojunction with boosted charge separation efficiency and promoted CO_2_ reduction activity. This work reports the design of photocatalysts with matched band gap structure and an intimate interface is an effective strategy to realize highly efficient CO_2_ reduction.

## Data Availability

The datasets presented in this study can be found in online repositories. The names of the repository/repositories and accession number(s) can be found in the article/Supplementary Material.
