# Supplementary material for: Construction of UiO-66/Bi4O5Br2 Type-II Heterojunction to Boost Charge Transfer for Promoting Photocatalytic CO2 Reduction Performance
Source: Front Chem. 2021 Dec 13;9:804204. doi: 10.3389/fchem.2021.804204 (PMC8710753; doi:10.3389/fchem.2021.804204)
Supplement: Supplementary file 1 [file DataSheet1.docx]

**Supporting Information**

**Construction of UiO-66/Bi_4_O_5_Br_2_ type-II heterojunction to boost charge transfer for promoting photocatalytic CO_2_ reduction performance**

*Dongsheng Li^a^, Bichen Zhu^a^, Zhongti Sun^a^, Qinqin Liu^a,^*^[[1]](#footnote-1)^**, Lele Wang^a^, Hua Tang^b*^*

^1^ School of Materials Science and Engineering, Jiangsu University, Zhenjiang, Jiangsu, 212013,

P. R. China

^2^ School of Environmental Science and Engineering, Qingdao University, Qingdao, 266071,

P. R. China


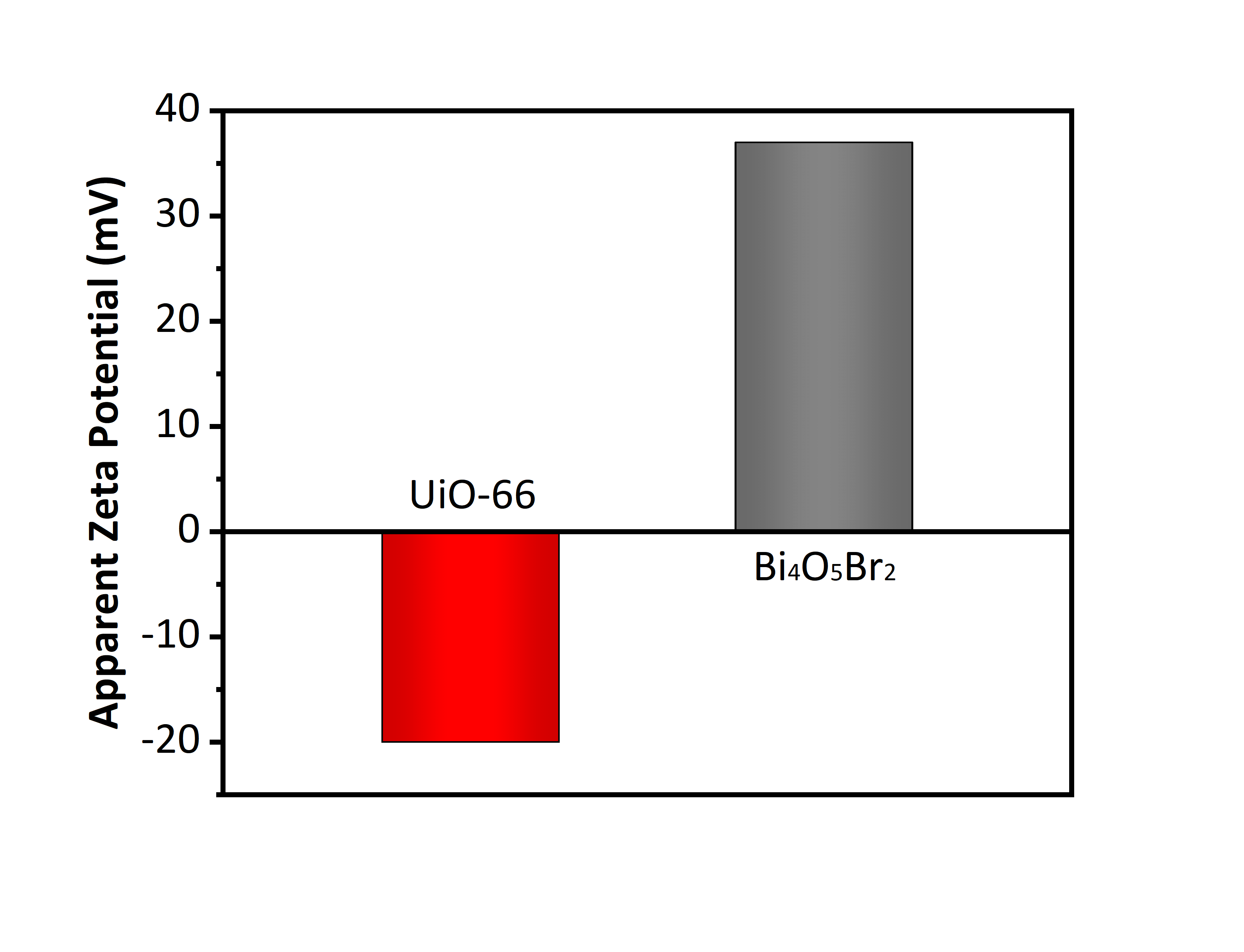


**Figure S1** Zeta potentials of UiO-66 and Bi_4_O_5_Br_2_.


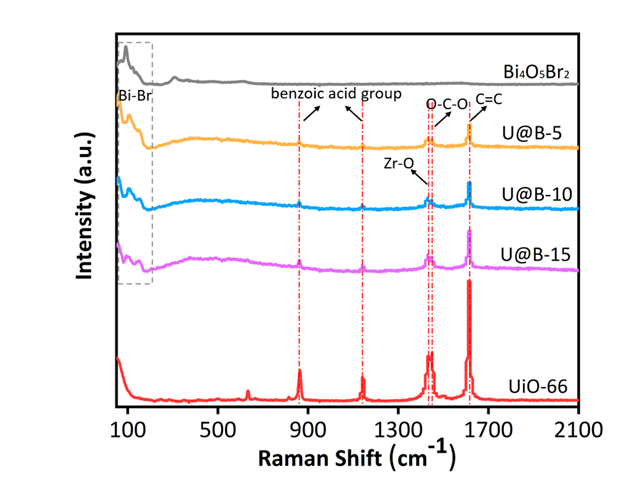


**Figure S2** Raman patterns of UiO-66, Bi_4_O_5_Br_2_ and U@B-X.


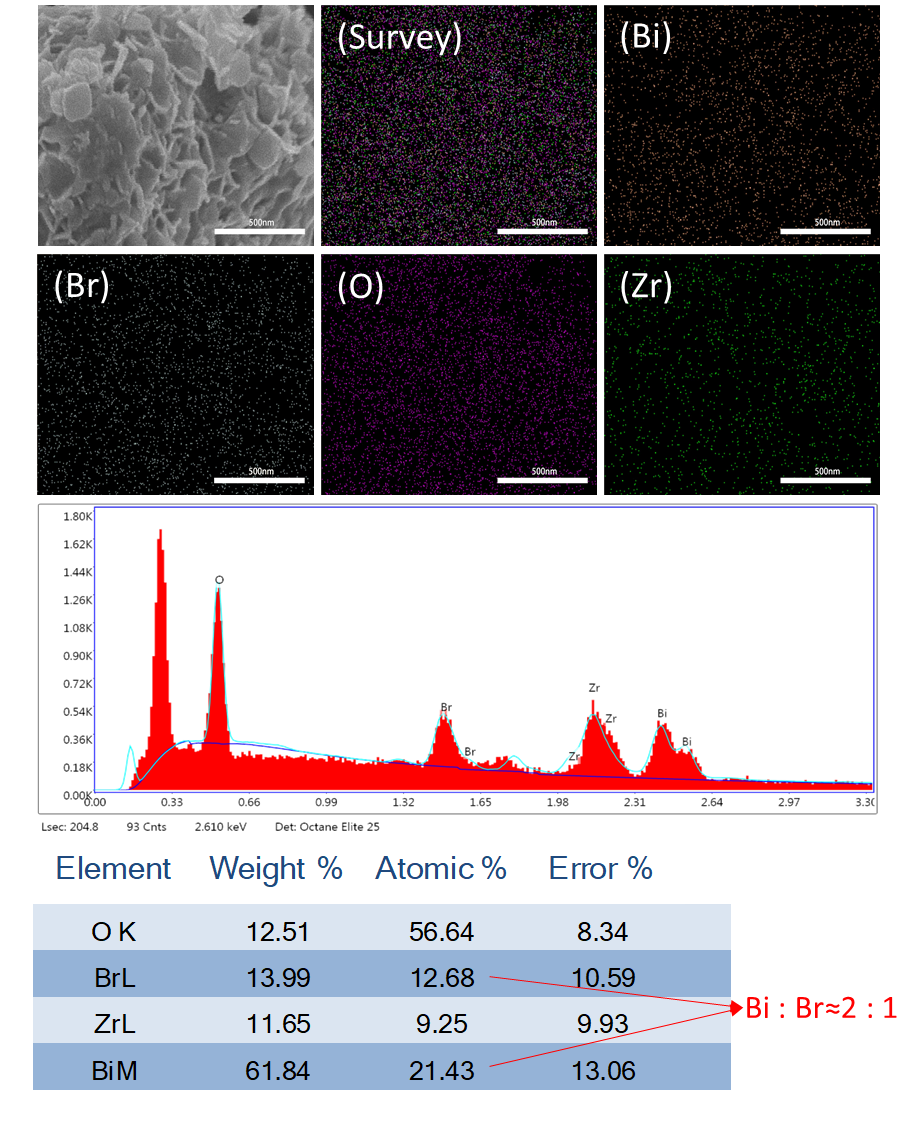


**Figure S3** Energy-dispersive X-ray spectroscopy (EDX) images of U@B-10.


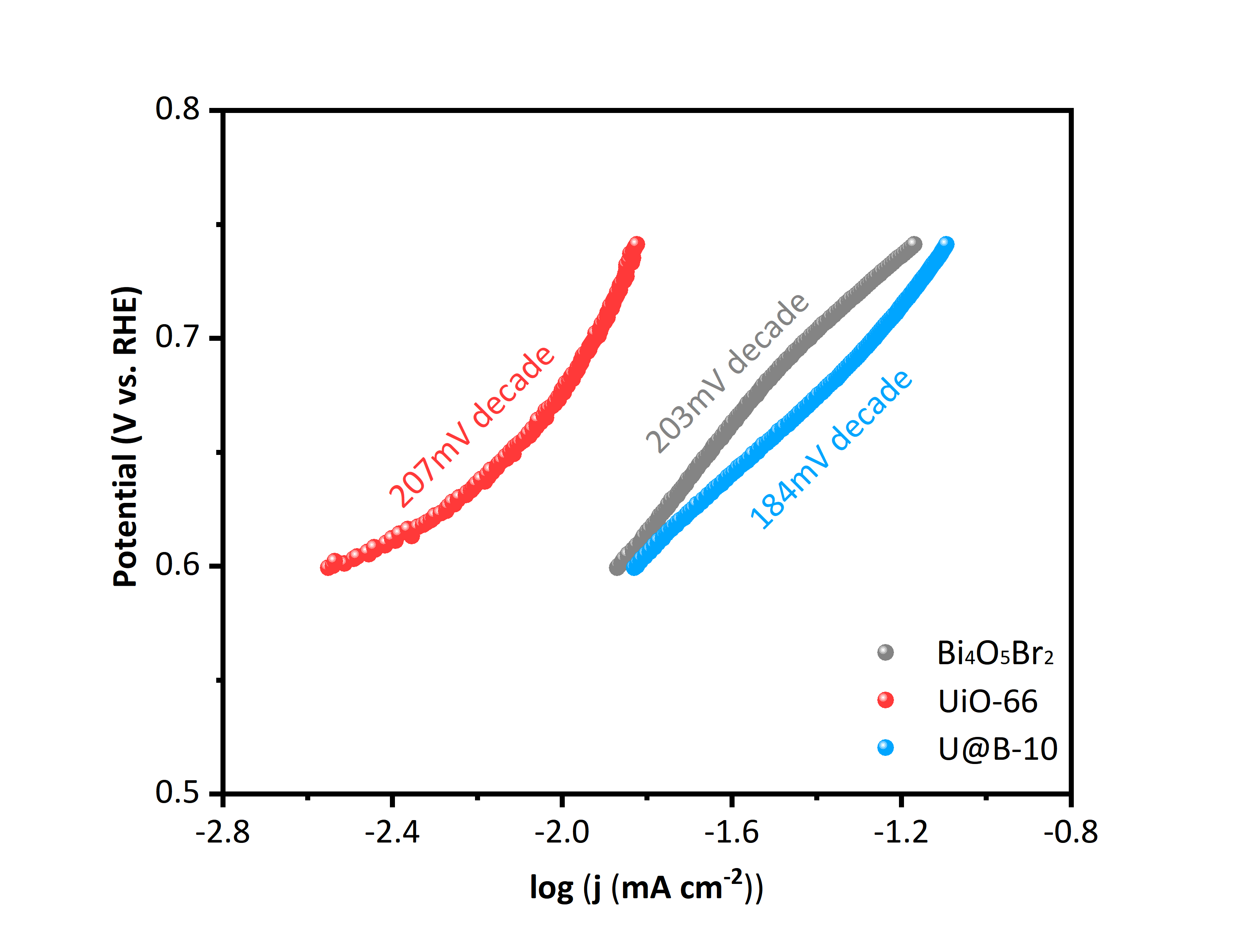


**Figure S4** Tafel slope plots**.**

**Table S1** BET surface area, pore volume and average pore diameter of pure UiO-66, Bi_4_O_5_Br_2_ and U@B-10 composite materials.

| **Sample** | **S_BET_ (m^2^ g^−1^)^a^** | | **Pore volume (cm^3^ g^−1^)^b^** | **Average pore size (nm)^c^** |
| --- | --- | --- | --- | --- |
| UiO-66 | 1353.49 | 0.522 | | 1.41 |
| Bi_4_O_5_Br_2_ | 22.62 | 0.049 | | 4.86 |
| U@B-10 | 211.12 | 0.142 | | 5.92 |

^a^ BET specific surface area, ^b^ Total pore volume calculated at P/P_0_ = 0.99,

^c^ Average pore size calculated using BJH method.

**Table S2** Comparison of CO_2_ reduction with other BiOX and UiO-66 based catalysts

| **Entry** | **Photocatalyst** | **Light sources** | **Reaction conditions** | **CO reduction rate (μmol·h^-1^·g^-1^)** | **Ref** |
| --- | --- | --- | --- | --- | --- |
| 1 | UiO-66/  Bi_4_O_5_Br_2_ | 300W xenon lamp | 5℃ CO_2_  Liquid-surface, water | 8.35 | This work |
| 2 | UiO-66/  Bi_2_S_3_ | 300W xenon lamp | 150℃ CO_2_  Gas-surface, water | 25.6 | [1] |
| 3 | Bi_4_O_5_Br_2_ | 300W xenon lamp | 20℃ CO_2_  Gas-surface, water | 2.73 | [2] |
| 4 | NH_2_-UiO-66/TiO_2_ | 150W xenon lamp | Gas-surface  CO_2_, H_2_ | 4.20 | [3] |
| 5 | BiPO_4_/BiOBr | 300W xenon lamp | 20℃ CO_2_  Gas-surface, water | 3.14 | [4] |
| 6 | Bi_2_O_4_/BiOBr | 300W xenon lamp | 20℃ CO_2_  Gas-surface, water | 2.6 | [5] |
| 7 | BiOCl | 500W  xenon lamp | Liquid-surface  CO_2,_ water | 1.01 | [6] |
| 8 | Bi_2_O_2_(OH)(NO_3_)-Br | 300W xenon lamp | 20℃ CO_2_  Gas-surface, water | 8.12 | [7] |
| 9 | BiOBr_0.6_Cl_0.4_ | 300W xenon lamp | 25℃ CO_2_  Gas-surface, water | 15.86 | [8] |

**References**

[1] X. Chen, Q. Li, J. Li, J. Chen, H. Jia, Modulating charge separation via in situ hydrothermal assembly of low content Bi_2_S_3_ into UiO-66 for efficient photothermocatalytic CO_2_ reduction, Appl. Catal. B Environ. 270 (2020) 118915.

[2] L. Ye, X. Jin, C. Liu, C. Ding, H. Xie, K.H. Chu, P.K. Wong, Thickness-ultrathin and bismuth-rich strategies for BiOBr to enhance photoreduction of CO_2_ into solar fuels, Appl. Catal. B Environ. 187 (2016) 281-290.

[3] A. Crake, K.C. Christoforidis, A. Kafizas, S. Zafeiratos, C. Petit, CO_2_ capture and photocatalytic reduction using bifunctional TiO_2_ /MOF nanocomposites under UV–vis irradiation, Appl. Catal. B Environ. 210 (2017) 131-140.

[4] J. Zhu, Y. Li, X. Wang, J. Zhao, Y. Wu, F. Li, Simultaneous phosphorylation and Bi modification of BiOBr for promoting photocatalytic CO_2_ reduction, ACS Sustainable Chem. Eng. 7 (2019) 14953-14961.

[5] D. Wu, L. Ye, S. Yue, B. Wang, W. Wang, H.Y. Yip, P.K. Wong, Alkali-induced in situ fabrication of Bi_2_O_4_-decorated BiOBr nanosheets with excellent photocatalytic performance, J. Phys. Chem. C 120 (2016) 7715-7727.

[6] L. Zhang, W. Wang, D. Jiang, E. Gao, S. Sun, Photoreduction of CO_2_ on BiOCl nanoplates with the assistance of photoinduced oxygen vacancies, Nano Res. 8 (2014) 821-831.

[7] L. Hao, L. Kang, H. Huang, L. Ye, K. Han, S. Yang, H. Yu, M. Batmunkh, Y. Zhang, T. Ma, Surface-halogenation-induced atomic-site activation and local charge separation for superb CO_2_ photoreduction, Adv. Mater. 31 (2019) 1900546.

[8] M. Gao, J. Yang, T. Sun, Z. Zhang, D. Zhang, H. Huang, H. Lin, Y. Fang, X. Wang, Persian buttercup-like BiOBr_x_Cl_1-x_ solid solution for photocatalytic overall CO_2_ reduction to CO and O_2_, Appl. Catal. B Environ. 243 (2019) 734-740.

1. * Corresponding author. Tel.: +86 13921581597; E-mail address: [qqliu@ujs.edu.cn](mailto:qqliu@ujs.edu.cn)

   * Corresponding author. E-mail address: [huatang79@163.com](mailto:huatang79@163.com) [↑](#footnote-ref-1)
